# Neurobehavioral Mechanisms Supporting Trust and Reciprocity

**DOI:** 10.3389/fnhum.2019.00271

**Published:** 2019-08-13

**Authors:** Dominic S. Fareri

**Affiliations:** Gordon F. Derner School of Psychology, Adelphi University, Garden City, NY, United States

**Keywords:** trust, social learning, computational modeling, reciprocity, social decision-making

## Abstract

Trust and reciprocity are cornerstones of human nature, both at the levels of close interpersonal relationships and economic/societal structures. Being able to both place trust in others and decide whether to reciprocate trust placed in us is rooted in implicit and explicit processes that guide expectations of others, help reduce social uncertainty, and build relationships. This review will highlight neurobehavioral mechanisms supporting trust and reciprocity, through the lens of implicit and explicit social appraisal and learning processes. Significant consideration will be given to the neural underpinnings of these implicit and explicit processes, and special focus will center on the underlying neurocomputational mechanisms facilitating the integration of implicit and explicit signals supporting trust and reciprocity. Finally, this review will conclude with a discussion of how we can leverage findings regarding the neurobehavioral mechanisms supporting trust and reciprocity to better inform our understanding of mental health disorders characterized by social dysfunction.

## Introduction

Virtually all aspects of human social life are based on trust and reciprocity. Trust is a multifaceted, socially risky construct (Krueger and Meyer-Lindenberg, 2019) that not only underlies the success of our business and economic structures but is also a pillar on which close relationships and social networks are built. We can conceptualize trust as a process based on *social expectations* (Cox, 2004), whereby we assume mutual risk with another person—e.g., business colleague, close friend—in collaboration toward a shared goal (Simpson, 2007); reciprocity (or betrayal) of trust, then, provides feedback that guides social learning (Fareri et al., in press). Continued reciprocity from others can be socially rewarding, and can fulfill basic social needs of support and belongingness (Baumeister and Leary, 1995), without which we are likely to experience poor physical, emotional, and mental health outcomes (Cacioppo et al., 2015; Eisenberger et al., 2017).

Decisions to place trust in others and reciprocate trust placed in us are rooted in both *implicit* social appraisals—i.e., rapid evaluations of others based on minimal information—and *explicit* social learning processes resulting from direct interpersonal experience or presentation of social information. Both types of processes (summarized in Table 1) facilitate learning about others and reduction of social uncertainty (FeldmanHall and Chang, 2018; Fareri et al., in press). This article will review evidence regarding how decisions to trust and reciprocate are informed by implicit and explicit processes, how updating social expectations may be influenced by social priors, and how social and reward-related neural circuits support trust and reciprocity. This article also discusses the utility of computational accounts in characterizing implicit and explicit influences on trust and reciprocity, which may have important implications for mental health conditions characterized by dysregulated social function.

**Table 1:** Implicit and explicit signals guiding trust and reciprocity.

	Trust	Reciprocity
Implicit	Facial characteristics Race bias In/out-group bias Prosocial orientation	Prosocial orientation Personality traits Pupil dilation
Explicit	Experienced reciprocity Experienced trust violation Moral character Reputational priors	Bestowed trust Risk associated with trust Prior experience of reciprocity Threat of punishment

## Decisions to Trust Others

Deciding to place trust in someone represents one component of prosocial behavior. Recent theories suggest that prosociality is an automatic, intuitive process (Zaki and Mitchell, 2013) driven by the likelihood of success it brings in day-to-day life (Rand et al., 2016). Thus on its own, placing trust in another might reflect an adaptive strategy to ensure positive social outcomes by broadcasting to other people that we have a reputation for being willing and reliable interaction partners (Berg et al., 1995; Jordan et al., 2016).

### Implicit Influences on Trust Decisions

Prosocial intuitions to trust others can be shaped by a number of implicit processes. Drawing on basic evolutionary threat detection mechanisms (Delgado et al., 2006; Lindström and Olsson, 2015), we are capable of estimating the trustworthiness of a stranger almost automatically (i.e., on the order of milliseconds), from relatively minimal perceptual information such as the precise configuration of an individual’s facial features (Todorov, 2008), as well as from assumed social group knowledge. These rapid social evaluations implicitly guide initial *social approach/avoidance* decisions (i.e., should I engage with this new person) in that they occur outside of conscious awareness (Willis and Todorov, 2006). Evidence from fMRI studies implicate the amygdala (Engell et al., 2007; Todorov et al., 2008), as well as the medial prefrontal cortex (mPFC) and the precuneus (Todorov et al., 2008), all of which have been implicated in representing different forms of social information (Amodio and Frith, 2006; Van Overwalle and Baetens, 2009; Stanley, 2016), in rapid evaluations of facial trustworthiness. These regions differentially encode trustworthiness information: separate amygdala subregions show both quadratic and negative linear associations with facial trustworthiness (Todorov et al., 2008; Rule et al., 2013; Freeman et al., 2014), while the mPFC and precuneus demonstrate heightened responses to moderately trustworthy faces (Todorov et al., 2008).

Social approach and avoidance signals are themselves subjects to implicit influence. Social heuristics (e.g., race/gender stereotypes) can shape judgments of others and decisions to trust them outside of conscious awareness. Racial bias can be particularly pervasive: an individual’s implicit bias (IAT; Greenwald et al., 1998) towards white partners (and away from black partners) positively correlates with the degree to which one invests with a white (vs. black) partner (Stanley et al., 2011). Interestingly, this pattern correlates with striatal activation, implicating this region in the implicit encoding of group-level reputation, while the degree to which people invest with black vs. white partners correlates positively with amygdala activation (Stanley et al., 2012), possibly suggesting an implicit representation of perceived threat or social risk.

Implicit influences on trust decisions can more generally be shaped by in-group vs. out-group biases that may be rooted in political affiliations (Rigney et al., 2018) or support for rival sports teams (Cikara et al., 2011), for instance. People tend to trust individuals who attend their university or are from the same country relative to those from rival universities or other countries (Hughes et al., 2017a,b). Interestingly, as with race, the difference in striatal activation when trusting in-group relative to out-group members significantly correlates with the degree of one’s in-group bias (Hughes et al., 2017a). This effect may be mitigated by the amount of time one has to process trusting an outgroup member, suggesting that overcoming outgroup biases may require more deliberative thought and neural mechanisms of cognitive control (Hughes et al., 2017a). Taken together, implicit signals can significantly and quickly shape choices to place trust in others.

### Explicit Influences on Trust Decisions

Our choices to trust others can also be guided by particularly diagnostic information about another person (Bhanji and Beer, 2013; Mende-Siedlecki et al., 2013), which we can acquire explicitly through direct experiences with others or from another source (e.g., rumor spread by another person). Initial choices to place trust in others are subsequently met with either reciprocity or a violation of trust. This direct experience of a social outcome (i.e., positive or negative) can then inform our representation of a partner’s reputation and whether we want to trust them in the future. As is the case with implicit representation of group-level reputation, the striatum encodes the reputation of individuals stronger responses in the striatum are elicited by experienced reciprocity (vs. violations) of trust during repeated interactions, and these neural signals shift temporally backward as we learn to anticipate that a partner will act in a trustworthy manner (King-Casas et al., 2005). Similar patterns of activation have been reported in the mPFC, with enhanced BOLD activation observed when trusting others during initial stages of partnership building that decreases once reputation has been learned (Krueger et al., 2007). Thus, direct experience of reciprocity serves as both a socially rewarding commodity (Phan et al., 2010) that fluctuates based on patterns of cooperation (Rilling et al., 2002; Phan et al., 2010) and an explicit social *learning* signal that can guide trust decisions.

Information about moral character—which can be inferred from how likely one would be to consider another’s welfare (i.e., deontological) relative to the bottom line outcome (i.e., consequentialist)—may be particularly diagnostic when deciding whether to trust a partner (Everett et al., 2016). Individuals endorsing deontological choices (i.e., would not endorse killing one person to save five) are consistently seen as more moral and trustworthy and are trusted more often in one-shot trust games (Everett et al., 2016, 2018). Information about moral character can create a persistent and outsized influence on our ability to encode explicit signals of reciprocation and defection of trust from another person. Learning that someone performed a selfless deed (e.g., risking their life for another person) can facilitate impressions (i.e., prior) of that person as highly moral and trustworthy, even if they happen to violate our trust at a later point, a process resembling confirmation bias (Doll et al., 2009). Strong moral impressions also modulate striatal function during repeated social interactions based on trust such that they eliminate the canonical social reward response in the striatum to reciprocity relative to defection, inhibiting learning *via* explicit experience (Delgado et al., 2005). Reputational priors may be encoded within the mPFC, as this region demonstrates increased activation when faced with a choice to trust a partner about whom priors exist (relative to those about whom we have no knowledge; Fouragnan et al., 2013). Thus, explicitly acquired social information can both incrementally shape our ability to learn to trust others while also inhibiting our ability to adapt to social interactions.

### Neurocomputational Mechanisms Supporting Trust Decisions

Implicit and explicit signals thus both play a role in decisions to trust. Increases in the use of computational modeling approaches (Kishida and Montague, 2012; Cheong et al., 2017) may shed light on the interaction between these different types of signals. One hypothesis suggests that a choice to place trust in another is not static, but rather dynamically evolves over time as we update initial implicit appraisals of others with explicitly experienced patterns of reciprocity (Chang et al., 2010) using associative mechanisms that enable learning the value of a partner on a trial-by-trial basis (Rescorla and Wagner, 1972). Importantly, this *dynamic belief* model of trustworthiness allows for: (1) differential weighting of reciprocity (or defection) as a function of the strength and valence of an initial impression of a partner; and (2) for initial impressions and experienced outcomes to influence each other in order to learn about a partner. In other words, whether a partner reciprocates or violates trust informs the updating of an impression, which then feeds forward to differentially weight subsequent instances of reciprocity/violation of trust (Chang et al., 2010).

Computational modeling also highlights different ways in which priors shape trust decisions. Learning to trust partners can be better explained by a model assuming that we learn differently about others (relative to a model assuming no social biases) based on whether we have priors about their tendency to cooperate (Fouragnan et al., 2013). Further, striatal representation of prediction error signals (i.e., increased activation when expectations do not match outcomes) is *absent* for those partners about whom instructed priors exist (Fouragnan et al., 2013), suggesting that priors shape behavior *via* top-down neural mechanisms. Other work demonstrates that people tend to weight and use reciprocity and violations of trust (as indexed by different learning rates) in ways that are consistent with prior impressions of others as “good” or “bad”: reciprocity from someone initially perceived as trustworthy contributes more heavily to subsequent choices to a violation of trust from that same partner, and* vice versa* (Fareri et al., 2012). Computational approaches have also tested competing hypotheses about whether trust decisions are motivated differently as a function of whether we have an existing relationship with a partner (Fareri et al., 2015). Modeling analyses revealed that choices to trust friends relative to strangers are driven not by stronger priors associated with a friend, but rather by differential weighting of experienced reciprocity as a function of social closeness with a partner (i.e., greater social value of reciprocation from a friend than from a stranger); this social weighting is encoded within the ventral striatum and mPFC (Fareri et al., 2015). Computational approaches thus demonstrate that implicit information (i.e., facial characteristics) and explicit information (i.e., social priors, relationship closeness) shape the way in which we use experienced reciprocity to inform choices to trust. It is worth noting that these computational processes allow for the possibility of explicitly acquired social information (i.e., moral information) to act implicitly in guiding neural and behavioral responses.

## Decisions to Reciprocate Trust

Whereas a choice to place trust in another person involves approach/avoidance mechanisms and a desire to signal a willingness to be cooperative, reciprocity involves higher-level considerations in conjunction with implicit appraisal processes. Reciprocity is necessarily more informed by individual differences in other-regarding preferences, more explicit person-level information—i.e., did this person place their trust in me?—and consideration of how others will react to our actions.

### Implicit Influences on Reciprocity

Reciprocity can be driven in part by inferences based on physiological signals from those that bestow trust upon us and from trait-level individual differences (Thielmann and Hilbig, 2015). For example, much like information from the eyes can inform us about potential threats in the environment (Kim et al., 2016), we can also use signals from the eyes to guide our choices to reciprocate trust. People tend to reciprocate trust from others who display more dilated pupils (Kret and De Dreu, 2019); increases in pupil dilation may reflect a desire for affiliation (though see also Fehr and Schneider, 2010). People may also be guided to reciprocate based on their *own tendencies* toward being prosocial (vs. self-interested), which may be quantified as the trade-off in value between outcomes for the self vs. outcomes for others (i.e., social value orientation; McClintock and Allison, 1989; Van Lange, 1999). Prosocial orientation positively correlates with amygdala activation when evaluating distributions of reward outcomes between self and other (Haruno and Fridth, 2009), suggesting this to be an implicit, internal process that may guide future choices in social interactions. People who tend to be more prosocial demonstrate greater levels of reciprocity overall and are more sensitive to a partner’s pattern of cooperation (Van Lange, 1999; van den Bos et al., 2009). These trait level differences tend to be reflected in the recruitment of neural circuits supporting social processes and cognitive control, such that acting contradictory to one’s typical orientation engages activation in the right temporoparietal junction (rTPJ), precuneus and dorsal anterior cingulate cortex (dACC; van den Bos et al., 2009).

### Explicit Influences on Reciprocity

Decisions to reciprocate trust necessarily involve evaluation of explicit social signals. The degree to which an interaction partner places trust in us informs whether we should feel inclined to reciprocate and whether we can expect that they will act similarly in the future. Bestowed trust may be perceived as a social reward signal that indicates something about reputation and shapes our own propensity to reciprocate. The striatum is implicated in encoding this type of explicit signal, and the degree to which these instances of trust are diagnostic of another person’s reputation is associated with temporal shifts in the striatal response to anticipating a partner’s decisions’ (King-Casas et al., 2005). Importantly, these choices to reciprocate are subject to contextual information. Knowledge of the risk another person may be taking by placing trust in us can shape our choices to engage in reciprocity. People tend to reciprocate more often when someone is taking a large chance of incurring a loss by trusting us; reciprocity in such high-risk situations is associated with increases in rTPJ recruitment (van den Bos et al., 2009). Choices to reciprocate are also positively correlated with the degree to which people have experienced reciprocity from others in the past (Cáceda et al., 2017). Furthermore, if explicitly threatened with a penalty (i.e., sanction) for not reciprocating trust, people tend to reciprocate to a lesser degree (associated with anticipatory activation in the vmPFC) suggesting an aversive effect of explicit threats in reciprocity and relationship building (Li et al., 2009).

### Neurocomputational Mechanisms Supporting Reciprocity Decisions

One hypothesis emerging from the idea that sensitivity to others’ outcomes can drive reciprocity is that people are inequity averse, and try to remedy inequitable outcomes or distributions of resources (Fehr and Schmidt, 1999; Tricomi et al., 2010). A related, but competing hypothesis regarding reciprocity posits that we often act to minimize feelings associated with disappointing another person by not meeting their expectations of us. This phenomenon, known as guilt aversion (Dufwenberg and Gneezy, 2000; Battigalli and Dufwenberg, 2007), requires considering not just our own intentions and interests, but also the assumed expectations that a partner has about our own behavior (i.e., second-order beliefs: our estimation of the likelihood someone thinks that we will reciprocate their trust). Computational approaches have proven to be quite useful in parameterizing motivations for reciprocity such as inequity and guilt aversion. As many studies examining these processes are structured through the lens of economic interactions (i.e., trust game), guilt on the part of a trustee has been conceptualized as the *difference* between: (1) the monetary amount that a trustee thinks that an investor would expect them to send back (i.e., second-order belief); and (2) the amount that the trustee actually sends back. This difference is weighted by a guilt aversion parameter which indexes sensitivity to feeling guilt (Chang et al., 2011). People tend to use their second-order beliefs to guide reciprocity decisions, with greater guilt sensitivity associated with increased recruitment of regions supporting social, emotional, and cognitive control processes (i.e., TPJ, insula, dACC, dorsolateral PFC; Chang et al., 2011). Inequity, on the other hand, can be parameterized as a more general sensitivity to unequal distributions of resources, regardless of whom is affected (i.e., absolute difference between outcomes for self vs. other). Reciprocity decisions motivated by inequity aversion seem to implicate value-based regions such as the amygdala and ventral striatum (Nihonsugi et al., 2015). Interestingly, people may opportunistically alternate between reciprocation based on inequity aversion or guilt aversion by also accounting for one’s own self-interest outcome. This moral strategy model accounts for such a possibility by including a social preference parameter, which indicates that people are acting out of self-interest when it is equivalent to 0, but are motivated by guilt aversion or inequity aversion when this term is negative or positive, respectively (van Baar et al., 2019). Thus, the application of computational models has helped to dissociate different mechanisms underlying reciprocity decisions.

## Clinical Implications and Future Directions

Interpersonal difficulties centered on trust and reciprocity are at the heart of a host of mental health conditions (Montague et al., 2012; Kishida and Montague, 2013; Stanley and Adolphs, 2013). It is thus useful to consider how breakdowns of implicit and explicit processes supporting such decisions may contribute to difficulties in social function. Borderline personality disorder (BPD) represents a condition characterized by unstable relationships and dysregulated affect (Stanley and Siever, 2010). Individuals with BPD tend to be biased towards suspicion of others, showing a greater likelihood than healthy controls to view others as untrustworthy, and reduced neural responses to untrustworthy faces in the insula and lateral PFC (Fertuck et al., 2019). Individuals with BPD are also unable to maintain reciprocity in repeated interactions over time (King-Casas et al., 2008), possibly driven by difficulty representing others’ intentions (Stanley and Siever, 2010; Meyer-Lindenberg et al., 2011). Future work may incorporate computational approaches to probe whether failures to maintain reciprocity in BPD are related to altered computations of guilt aversion, or to the integration of beliefs about others’ intentions as well as the changes (i.e., volatility) of others’ behavior (Behrens et al., 2008; Diaconescu et al., 2014; Siegel et al., 2018).

Difficulty with appraising trustworthiness and placing trust in others are hallmarks of post-traumatic stress disorder (PTSD), which may underlie the propensity for re-victimization in this population (Fertuck et al., 2016). For example, individuals with PTSD are more likely to appraise unfamiliar faces as trustworthy compared to healthy controls, suggesting an impaired ability to integrate trust-related facial signals (Todorov et al., 2008). Further, women with PTSD associated with prior sexual assault show significantly lower levels of investment over time with partners and a decreased ability to learn partner reputation (i.e., lower learning rates in a computational model) in an economic trust game compared with healthy controls (Cisler et al., 2015). The application of Bayesian learning approaches, which can capture the ability to flexibly and dynamically update representations of moral character (Siegel et al., 2018), may be useful in further elucidating aberrant social learning processes in PTSD.

While the literature reviewed here highlights the utility of computational approaches to understanding mechanisms (i.e., guilt aversion, moral opportunism, relationship value) involved in trust and reciprocity, our understanding of neural and behavioral mechanisms of trust and reciprocity will be bolstered by integration with advanced neuroimaging approaches. Recent efforts linking individual differences in computational strategies supporting reciprocity (i.e., guilt aversion vs. moral opportunism) with differential patterns of brain activity assessed by inter-subject representational similarity analysis (van Baar et al., 2019) provide one template for combining computational and advanced imaging techniques. Other neuroimaging advances involve the use of network-level resting-state and task-based connectivity approaches to characterizing neural dynamics (Smith et al., 2009, 2014; Utevsky et al., 2017). Combining network connectivity approaches with computational modeling of behavior can help characterize how communication within and between neural networks supporting social processes and decision-making (e.g., default mode network, executive control network) are involved in the computation of implicit and explicit signals supporting trust and reciprocity. Such work may provide deeper and differential insight into neurocomputational breakdowns in trust across mental health conditions.

## Author Contributions

DF wrote the manuscript.

## Conflict of Interest Statement

The author declares that the research was conducted in the absence of any commercial or financial relationships that could be construed as a potential conflict of interest.
